# Adverse Events in Home Care: Identifying and Responding with interRAI Scales and Clinical Assessment Protocols

**DOI:** 10.1017/S0714980817000538

**Published:** 2018-03

**Authors:** Chi-Ling Joanna Sinn, Raquel Souza Dias Betini, Jennifer Wright, Lorri Eckler, Byung Wook Chang, Sophie Hogeveen, Luke Turcotte, John P. Hirdes

**Affiliations:** 1 School of Public Health and Health Systems, University of Waterloo; 2 Central Local Health Integration Network, Richmond Hill, ON; 3 University Health Network, Toronto, ON

**Keywords:** aging, caregivers, case management, decision support, home care, interRAI, vieillissement, aidants, gestion de cas, soutien à la décision, soins à domicile, interRAI

## Abstract

Outcomes of adverse events in home care are varied and multifactorial. This study tested a framework combining two health measures to identify home care recipients at higher risk of long-term care placement or death within one year. Both measures come from the Resident Assessment Instrument-Home Care (RAI-HC), a standardized comprehensive clinical assessment. Persons scoring high in the Method for Assigning Priority Levels (MAPLe) algorithm and Changes in Health, End-stage disease, Signs and Symptoms (CHESS) scale were at the greatest risk of placement or death and more than twice as likely to experience either outcome earlier than others. The target group was more likely to trigger mood, social relationship, and caregiver distress issues, suggesting mental health and psychosocial interventions might help in addition to medical care and/or personal support services. Home care agencies can use this framework to identify home care patients who may require a more intensive care coordinator approach.

## RÉSUMÉ

Les conséquences associées aux événements indésirables dans les soins à domicile sont variées et multifactorielles. La présente étude visait à tester un système d’évaluation joignant deux outils de mesures dans le but d’identifier les bénéficiaires de soins à domicile dont le risque de placement dans un établissement de soins de longue durée ou de décès dans l’année suivante est plus élevé. Les deux outils de mesure sont tirés du Système d’évaluation d’interRAI (Resident Assessment Instrument-Home Care; RAI-HC), un outil d’évaluation standardisé et détaillé. Les personnes présentant des scores élevés à l’algorithme de la Méthode d’attribution des niveaux de priorité (Method for Assigning Priority Levels; MAPLe) et à l’Échelle de mesure des changements de l’état de santé, des maladies en phase terminale, des signes et des symptômes (Changes in Health, End-stage disease, Signs and Symptoms; CHESS) étaient plus à risque de placement ou de décès, et leur risque de subir ces événements plus tôt comparativement aux autres patients était plus que doublé. Le groupe cible était aussi plus susceptible de présenter des problèmes d’humeur et de relations sociales, et leurs aidants risquaient davantage de souffrir de détresse, ce qui indiquerait que ces personnes et leurs aidants pourraient avoir besoin de soins de santé mentale et d’interventions psychosociales, en plus des soins médicaux et des services de soutien personnels. Les agences de soins à domicile pourraient utiliser ce système d’évaluation pour identifier les patients en soins prolongés à la maison chez qui une approche de soins coordonnés plus intensive serait nécessaire pour mieux atténuer et gérer les risques liés à leur condition.

One out of every six Canadians over age 65 receives formal home care services that may include personal support services, homemaking services, nursing, occupational therapy, physiotherapy, social work, speech language pathology, and nutritional counselling (Canadian Home Care Association, 2013). The national average reflects the use of home care services in Ontario, Quebec, and the Prairie provinces, whereas the ratio is lower in British Columbia, Nova Scotia, and Newfoundland and Labrador. Among Ontario recipients of long-stay home care, 97 per cent also receive support from an unpaid caregiver who may be a family member, friend, or neighbour; however, one third of caregivers express feelings of distress, anger, or depression or being unable to continue providing care (Health Quality Ontario, 2015). Rates of distress are higher among caregivers who care for persons with greater functional and cognitive impairment and frailty (Health Council of Canada, 2012; Health Quality Ontario, 2016). In 2009–2010, the proportion of long-stay home care patients who were classified as having high needs was 37 per cent (Ontario Association of Community Care Access Centres, 2015). The proportion of long-stay home care patients with high needs was 69 per cent in 2014–2015 and is expected to continue to rise. As the aging population continues to grow substantially over the next 10 years, providers of home care services must anticipate the needs of an increasingly complex group of home care recipients as well as the needs of their caregivers (Health Quality Ontario, 2016).

The published literature on outcomes of adverse events in home care focuses on risk for long-term care (LTC) placement and death, and suggests they are driven by different factors. A systematic review found that LTC placement was consistently predicted by functional impairment, cognitive impairment, and the inability of formal and informal care to meet daily living needs (Luppa et al., 2010). Mortality has been shown to be strongly associated with the Changes in Health, End-stage disease, Signs and Symptoms (CHESS) scale, Karnofsky Performance Scale, and Palliative Performance Scale (de Miguel Sánchez et al., 2006; Hirdes, Poss, Mitchell, Korngut, & Heckman, 2014; Lingjun, Jing, Jian, Wee, & Jijun, 2009; Mercadante et al., 2013). Individual risk factors for death include functional impairment, appetite loss, dysphagia, dyspnea, nutritional problems, poor self-rated health, poor quality of life, and recent hospitalization (Chernew, Weissert, & Hirth, 2001; Gené Badia et al., 2013; Kitamura, Nakamura, Nishiwaki, Ueno, & Hasegawa, 2010; Landi et al., 2012; Lingjun et al., 2009; Ostbye, Steenhuis, Wolfson, Walton, & Hill, 1999). Armstrong, Stolee, Hirdes, and Poss (2010) tested the potential utility of three conceptualizations of frailty for identifying home care recipients at risk of either LTC placement or death. Using the CHESS scale, Edmonton Frailty Scale (EFS), and Frailty Index (FI), the study authors were able to identify medium- and high-risk groups, but large amounts of unexplained variance remained in each model. The idea that outcomes of adverse events in home care are varied and multifactorial suggests that a single scale or composite score will have insufficient explanatory power for all outcomes of interest. Alternatively, a framework that brings together two or more scales capturing different indicators for LTC placement and death may be more useful than a single measure for identifying those in need of the highest level of care. Identifying this group of patients is paramount for developing tailored care plans that may avoid the outcome (i.e., LTC placement or death) or, when the outcome is unavoidable, ensure that the patient and family receive appropriate care during the transition process.

## High Risk Strategy Project

In Ontario, publicly funded home care services are coordinated by 14 Local Health Integration Networks (LHINs; formerly the Community Care Access Centres [CCACs]) that are local agencies established by the Ministry of Health and Long-Term Care. In 2012, Central LHIN embarked on the High Risk Strategy project. As part of this patient safety initiative, a team of Central LHIN staff reviewed over 1,700 client records for patient safety and risk events. In collaboration with graduate students from the University of Waterloo, the project sought to develop a simple decision support tool to proactively identify those at the greatest risk of LTC placement or death who would benefit from an adjustment or review of their care plan. When used in conjunction with existing interRAI tools, the proposed tool should highlight specific patient and caregiver needs and prompt care coordinators to provide resources that may mitigate the risk of an event, or at least ease the transition for patients and families. The goal of the project was to facilitate appropriate care planning for those at high risk that could be applied to the more than 36,000 long-stay home care patients who actively receive Central LHIN services on any given day.

The Resident Assessment Instrument-Home Care (RAI-HC) instrument is a comprehensive, standardized clinical assessment used widely in Canada and internationally (Canadian Home Care Association, 2013; Carpenter & Hirdes, 2013; Hirdes, Mitchell, Maxwell, & White, 2011). In Ontario, all adult, non-palliative home care patients who are expected to receive services for more than 60 days are assessed with the RAI-HC on admission and every six to 12 months or when there is a significant change in the patient’s health status. In addition to the assessment instrument, the RAI-HC assessment system includes outcome scales and decision support algorithms. The Method for Assigning Priority Levels (MAPLe) algorithm predicts LTC placement, caregiver distress, and ratings by the patient and/or caregiver that the patient would be better off in another living environment (Hirdes, Poss, & Curtin-Telegdi, 2008). The five MAPLe levels, ranging from very low to very high priority, are currently used by LHINs to determine eligibility, priority, and allocation of home care services. The CHESS scale ranges from zero to five, where high CHESS levels have been independently associated with greater likelihood of an adverse event and greater risk of mortality in home care populations (Doran et al., 2013; Hirdes, Frijters, & Teare, 2003; Hirdes et al., 2014). Early analyses established MAPLe and CHESS as the strongest predictors of LTC placement and death, respectively, and that independent variables not already included in the scales provided little additional explanatory power. Based on these analyses, and in the interest of producing a simple tool with existing outputs, the present study focused on identifying a “high risk” target group using the intersection of CHESS and MAPLe levels.

In addition to calculating scales that describe health status, the RAI-HC produces Clinical Assessment Protocols (CAPs) that alert the assessor to specific clinical, functional, cognition, mental health, and social life issues that are amenable to clinical intervention (Morris et al., 2010). The issues may be present at the time of assessment or at risk of developing in the future. Each CAP consists of four parts: a description of the issue; goals of care; a list of items that “trigger” the CAP; and care guidelines. Some CAPs have two levels of triggering to identify patients who have a higher than expected likelihood of declining and those who have an increased likelihood of improving. If a CAP is triggered, the care guidelines help the assessor to think through the relevant underlying issues and suggest strategies to resolve the problem, reduce the risk of decline, or increase the potential for improvement. The development and utility of CAPs in the interRAI assessment system have been discussed elsewhere (Freeman et al., 2014; Martin et al., 2009; Mathias, Hirdes, & Pittman, 2010; Neufeld, Perlman, & Hirdes, 2012).

This article illustrates the utility of using CHESS and MAPLe to identify patients at the highest risk for LTC placement or death based on event rates, time to event, and triggering rates of CAPs. The article also provides guidance on how to realize the practical value of CAPs for guiding care plans of high-risk patients.

## Methods

### Sample

Data used in this study were sent to University of Waterloo by Health Shared Services Ontario (HSSOntario; formerly the Ontario Association of Community Care Access Centres (OACCAC)) through a license agreement between the two organizations. The use of data from all LHINs provides a better representation of the entire province, where the results can be generalized across Ontario and not just to one LHIN. Additional sensitivity analysis was done with Central LHIN data only to ensure that the results agreed with the provincial data. Ethics clearance was received from the Office of Research Ethics at the University of Waterloo (ORE# 18228).

The sample included all Ontario patients admitted to home care services who were expected to require services for more than 60 days, and assessed with the RAI-HC instrument from January 2010 to August 2014. Patients who were assessed in hospital, waiting for LTC placement, or assigned to a palliative home care team, were excluded from the analyses. The first RAI-HC assessment completed within 90 days of the referral date was selected for each patient (*n* = 242,923). A validation dataset was created by taking the cross-section of RAI-HC assessments done from January to December 2014 that was not limited to intake assessments only (*n* = 102,378).

### Definition of Target Group

Table 1 summarizes the distribution of CHESS and MAPLe levels in the sample. The cut-points chosen for this study were based on a request from Central LHIN that the target group comprise approximately 10 per cent of the total sample. The target group was defined as patients in both CHESS levels three to five (“high CHESS”) and MAPLe levels four to five (“high MAPLe”), accounting for 11.2 per cent of the sample. Similar proportions were seen across regional LHINs and ranged from 8.9 per cent to 15.0 per cent. The other groups included long-stay home care patients with high CHESS and low MAPLe (9.7%), long-stay home care patients with low CHESS and high MAPLe (30.0%), and those meeting neither of the criteria (49.2%).

**Table 1:** Distribution of CHESS and MAPLe levels

% (*n*) CHESS	MAPLe				
	1	2	3	4	5
0	6.2 (15,135)	1.5 (3,677)	3.8 (9,322)	4.8 (11,555)	1.2 (3,013)
1	6.2 (15,107)	3.5 (8,554)	11.2 (27,254)	8.4 (20,470)	2.7 (6,453)
2	3.6 (8,816)	2.6 (6,373)	10.4 (25,268)	8.7 (21,227)	4.1 (10,027)
3	1.2 (3,013)	1.3 (3,160)	6.1 (14,754)	5.5 (13,403)	2.4 (5,708)
4	0.04 (97)	0.04 (90)	0.9 (2,269)	2.0 (4,830)	1.2 (2,811)
5	– (0)	– (0)	0.04 (109)	0.1 (302)	0.1 (126)

CHESS = Changes in Health, End-stage disease, Signs and Symptoms

MAPLe = Method for Assigning Priority Levels

### Definition of Outcomes

Referral and discharge information were obtained from the Client Health and Related Information System (CHRIS), a web-based system used by all Ontario LHINs. For the survival analysis, the number of days following the assessment was calculated as the difference between the RAI-HC assessment date and discharge date, and was censored after 365 days. Discharge codes were used to determine the occurrence of either LTC placement or death.

### Statistical Analysis

Details about the development of MAPLe and CHESS and the clinical variables covered by each algorithm can be found in Hirdes, Poss, and Curtin-Telegdi (2008) and Hirdes, Frijters, and Teare (2003). χ^2^ tests for independence and post hoc tests were used to examine the distributions of socio-demographic, clinical, and other health characteristics, as well as the rate of outcomes among the defined groups. Positive predictive value and negative predictive values were also calculated. Time to event was examined using Cox proportional hazards regression. Analyses were done at the provincial level as well as for each regional LHIN. Results at both levels were very similar, so only the provincial results are reported in this article. All statistical analyses were done using SAS, Version 9.4 (SAS Institute Inc.).

## Results

Compared to other long-stay home care patients, the target group had more males and persons over age 75, and fewer lived alone (Table 2). Cut-off scores of three or greater on the Activities of Daily Living Hierarchy Scale, Instrumental Activities of Daily Living Capacity Scale, and Cognitive Performance Scale were used to summarize functional and cognitive status. More patients in the target group had moderate to severe impairment in basic activities of daily living (ADLs; i.e., extensive or greater assistance needed with at least one ADL) as well as in instrumental activities of daily living (IADLs; i.e., some assistance needed with all IADLs). The proportion of patients with moderate to severe cognitive impairment was three times greater in the target group compared to other long-stay home care patients.

**Table 2:** Socio-demographic, clinical, and other health characteristics of long-stay home care recipients by CHESS and MAPLe level

Characteristic		High CHESS and High MAPLe, % (*n*)	All Other Home Care Recipients, % (*n*)	*p*
Female		55.6 (15,122)	60.4 (130,208)	<.0001
Age	<65	11.4 (3,102)	18.1 (39,137)	<.0001
	65–75	15.7 (4,263)	17.5 (37,754)	
	75–85	37.7 (10,241)	35.2 (75,926)	
	≥85	35.2 (9,574)	29.2 (62,926)	
Married		43.9 (11,943)	43.7 (94,311)	.48
Lived alone		29.3 (7,971)	32.2 (69,546)	<.0001
Moderate to severe ADL impairment^a^		22.3 (6,065)	9.9 (21,262)	<.0001
Moderate to severe IADL impairment^b^		92.2 (25,064)	74.9 (161,568)	<.0001
Moderate to severe cognitive impairment^c^		24.8 (6,728)	8.1 (17,558)	<.0001
Cancer diagnosis		8.9 (2,410)	8.0 (17,558)	<.0001
Proportion of total sample		11.2 (27,180)	88.8 (215,743)	

a Activities of Daily Living Hierarchy Scale 3–6 (total scale ranges from 0–6).

b Instrumental Activities of Daily Living Capacity Scale 3–6 (total scale ranges from 0–6).

c Cognitive Performance Scale 3–6 (total scale ranges from 0–6).

Table 3 provides the rates of discharge from home care service due to LTC placement and death, separately and combined, for the four intersecting groups of CHESS and MAPLe. Within one year, the proportion of home care patients who had died was highest in the target group (15.2%) and among patients with high CHESS and low MAPLe (17.0%). Similarly, the proportion of patients who were placed into LTC were highest in the target group (10.9%) and among those with low CHESS and high MAPLe (9.6%). When the risk of LTC placement and death were combined into a single outcome, 26.1 per cent of the target group experienced either outcome within one year, and the post hoc tests confirmed that this rate was significantly higher than in any other group. The positive predictive value was 26.1 per cent and the negative predictive value was 88.5 per cent.

**Table 3:** Rate of outcomes of adverse events among long-stay home care recipients by CHESS and MAPLe level within one year of assessment

Outcome of adverse events	High CHESS and high MAPLe, % (*n)* 11.2 (27,180)	High CHESS and low MAPLe, % (*n*) 9.7 (23,492)	Low CHESS and high MAPLe, % (*n*) 30.0 (72,745)	Low CHESS and low MAPLe, % (*n*) 49.2 (119,506)	*p*
Discharge from home care service due to LTC admission	10.9 (2,963)	3.0 (707)	9.6 (6,958)	2.0 (2,325)	<.0001
Discharge from home care service due to death	15.2 (4,138)	17.0 (3,989)	5.4 (3,923)	5.8 (6,921)	<.0001
Discharge from home care service due to LTC admission or death	26.1 (7,101)	20.0 (4,696)	15.0 (10,881)	7.7 (9,246)	<.0001

**Figure 1:**
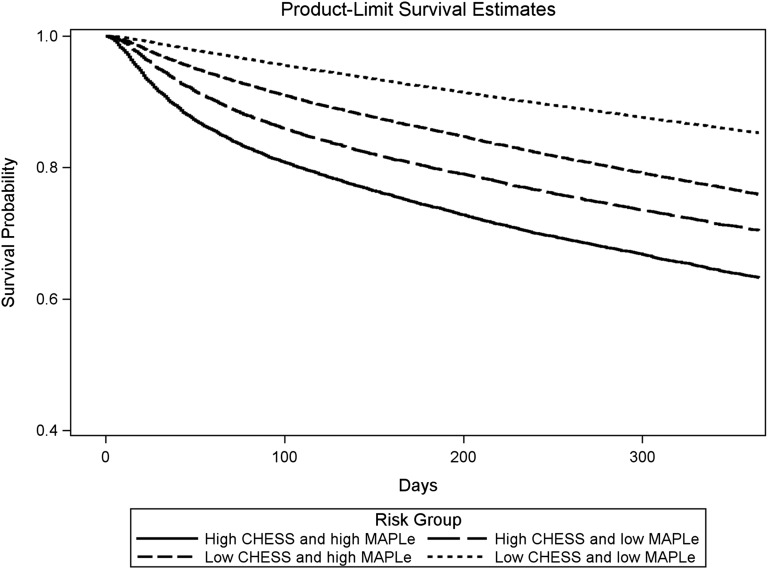
Time to outcomes of adverse events among long-stay home care recipients by CHESS and MAPLe level

The survival curves from Figure 1 further distinguish the target group from the other groups by incorporating time-to-event data. All four curves follow a similar negative linear shape, but the target group showed the poorest survival probability. When all other groups were combined, patients in the target group were more than twice as likely to be placed into LTC or die earlier at any point in time (hazard ratio [HR] = 2.4, *p* < .0001). Alternatively stated, patients in the target group had a 71 per cent chance of experiencing either outcome earlier than those in other groups (probability = HR / (HR + 1) = 2.4 / 3.4).

Table 4 lists the most frequently triggered CAPs among patients with high CHESS or high MAPLe. The purpose of showing these scales separately is to illustrate their individual value related to the CAPs. Both groups triggered the CAPs for ADL (facilitate improvement), cognitive loss (prevent decline), falls, mood, physical activity promotion, and urinary incontinence. Patients with high CHESS tended to trigger the CAPs for appropriate medication, cardiorespiratory symptoms, and pain, whereas those with high MAPLe triggered the CAPs for behaviour and communication (prevent decline) at a higher rate. Since the same items are built into the algorithms (e.g., ADL and cognition measures), it is unsurprising to find that their matching CAPs are more likely to be triggered. It is of greater interest to note the triggering patterns of CAPs using items not built into the algorithms that are highlighted in Table 5. Although the rates of triggering the mood and social relationships CAPs are similar between the high CHESS and low MAPLe group and low CHESS and high MAPLe group, these CAPs were triggered by a significantly greater proportion of patients in the target group compared to any other group. The same pattern was observed with rates of caregiver distress. In all cases, patients with low CHESS and low MAPLe were the least likely to trigger these areas of risk.

**Table 4:** Clinical Assessment Protocols (CAPs) triggered among long-stay home care recipients with high CHESS or high MAPLe levels

CAP	Trigger Level	High CHESS only, % (*n*)	High MAPLe only, % (*n*)
ADL	Facilitate improvement	49.7 (25,172)	41.4 (41,384)
Appropriate medication	Triggered	48.0 (24,337)	23.7 (23,712)
Behaviour	Triggered	12.2 (6,150)	20.8 (20,833)
Cardiorespiratory	Triggered	78.1 (39,596)	44.0 (44,006)
Cognitive loss	Prevent decline	40.1 (20,303)	34.3 (34,302)
Communication	Prevent decline	14.3 (7,227)	21.4 (21,399)
Falls	Medium risk	23.7 (12,022)	18.9 (18,843)
	High risk	26.6 (13,456)	35.3 (35,227)
Mood	Low risk	29.2 (14,811)	28.0 (27,978)
	High risk	31.1 (15,778)	27.0 (27,013)
Pain	Medium priority	44.1 (22,356)	37.0 (36,992)
	High priority	19.6 (9,929)	12.3 (12,313)
Physical activity promotion	Triggered	39.5 (19,992)	31.3 (31,307)
Urinary incontinence	Prevent decline	19.9 (10,088)	27.6 (27,623)
	Facilitate improvement	23.6 (11,975)	14.4 (14,432)
Proportion of total sample		20.9 (50,669)	41.1 (99,924)

**Table 5:** Selected areas of heightened risk among persons with high CHESS and high MAPLe levels

Clinical Issue	High CHESS and high MAPLe, % (*n*) 11.2 (27,180)	High CHESS and low MAPLe, % (*n*) 9.7 (23,492)	Low CHESS and high MAPLe, % (*n*) 30.0 (72,745)	Low CHESS and low MAPLe, % (*n*) 49.2 (119,506)	*p*
Mood CAP (high risk)	37.0 (10,065)	24.3 (5,713)	23.3 (16,948)	14.3 (17,102)	<.0001
Social relationships CAP	17.0 (4,632)	13.4 (3,158)	13.2 (9,569)	11.0 (13,193)	<.0001
Caregiver expresses distress or is unable to continue in caring activities	48.8 (13,254)	28.3 (6,656)	36.4 (24,491)	15.9 (18,999)	<.0001

## Discussion

The intersection of high CHESS and high MAPLe is a useful and parsimonious method of identifying home care patients at risk of LTC placement or death. One in four long-stay home care patients in the target group will be placed into LTC or die within one year. Separately, persons with high CHESS were at greater mortality risk, supporting the fact that high CHESS, regardless of other variables captured in MAPLe, is associated with higher mortality whereas those with high MAPLe were at greater risk of LTC placement. There was a strong relationship between the variables used in the algorithms and the domains represented by the triggered CAPs among those with high scale scores. Both CHESS and MAPLe incorporate functional and cognition measures. Accordingly, patients who scored high either in CHESS or MAPLe were more likely to trigger the ADL and cognitive loss CAPs. Similarly, behaviours and falls are items used in the MAPLe algorithm, so patients with high MAPLe were more likely to trigger the corresponding CAPs. Issues related to ADL and cognition are strong predictors of LTC placement and are part of Ontario’s home care eligibility criteria (Gaugler, Yu, Krichbaum, & Wyman, 2009; Government of Ontario, 2010; Wattmo, Wallin, Londos, & Minthon, 2011). Implementation of strategies to reduce the risk of decline or increase the potential for improvement in ADL and cognition may have clinically significant positive effects on individual patients. The finding that half of the total home care sample (50.8%) had at least one high scale score is notable because it raises common clinical issues that warrant increased awareness on the part of health care providers. In addition to the aforementioned clinical issues, the target group showed higher rates of the mood CAP, social relationships CAP, and caregiver distress. These findings suggest that addressing mental health and psychosocial issues of both patients and caregivers may be particularly important for this high-risk group.

Mental health and psychosocial issues are difficult to disentangle and often occur together. In the general home care population, the prevalence of depressive symptoms and major depression is estimated to be 11–16 per cent and 6–14 per cent (Bruce et al., 2002, 2004; Ell, Unützer, Aranda, Sanchez, & Lee, 2005; Phillips et al., 1997; Pickett, Raue, & Bruce, 2012). Depressive symptoms and depression have been shown to be independent predictors of LTC placement and death even after controlling for socio-demographic factors and co-morbidities (Harris, 2007; Harris & Cooper, 2006; Schulz et al., 2000). Nearly one third of patients aged 65 and older living in private homes have reported low positive social interaction (Gilmour, 2012). Among older adults, increasing age is also associated with greater social vulnerability (Keefe, Andrew, Fancey, & Hall, 2006). There is growing evidence that social engagement is a promising intervention target for the treatment of depressive symptoms in older adults (Glass, De Leon, Bassuk, & Berkman, 2006; Isaac, Stewart, Artero, Ancelin, & Ritchie, 2009; Stoeckel & Litwin, 2015). Isaac et al. (2009) found that high social activity was the main factor predicting improvement in baseline depressive symptoms over a two-year follow-up. Among older adults in primary care settings, social interaction weakened the association between illness burden and depression symptoms (Hatfield, Hirsch, & Lyness, 2013). Regardless of whether depression is the cause of, or a marker for, life-threatening conditions, the purpose of the CAPs is to prompt the assessor to review and address underlying issues that place the individual at increased risk.

Informal caregivers provide essential support to enable their family members or friends to continue living at home. Although caregiving is associated with numerous benefits, the majority of informal caregivers report challenges to their physical and emotional health as well as their work-life balance (Turner & Findlay, 2012). This study’s findings are in line with previous work that showed significantly higher rates of caregiver distress as MAPLe levels increase, with up to 40–50 per cent of caregivers of patients in the very high MAPLe level showing distress (Canadian Home Care Association, 2013). Additionally, the present findings show that the addition of high CHESS strengthens the ability to identify patients at high risk by controlling for health instability that is not explained by high MAPLe. The rates of caregiver distress are estimated to be two to four times greater for caregivers of patients with difficulties in key areas of everyday function, including physical functioning, comprehension, and communication (Canadian Institute for Health Information, 2010; Health Quality Ontario, 2016). In addition to meeting increased care demands, caregiving at the end of life can introduce other stressors in the form of feelings of helplessness, vulnerability, and anxiety (Milberg, Strang, & Jakobsson, 2004; Stajduhar, 2013). Studies have shown that depression and social isolation are common among these caregivers, often at similar rates as those for whom they provide care, suggesting that psychosocial and mental health challenges are important issues for both patients and caregivers (Boyd et al., 2004; Grunfeld et al., 2004). Caregiver distress and related concepts, such as feeling trapped in the caregiving role and dissatisfaction with life, have been shown to be highly associated with earlier LTC admission for patients with dementia (Gaugler, Kane, Kane, Clay, & Newcomer, 2003; Yaffe et al., 2002). These studies show that caregiver characteristics and their ability to cope with caregiving responsibilities must be considered when designing patient care plans.

### Implementation by Central LHIN

This framework will improve the prioritization of patients and mitigate risk before poor outcomes occur. The use of existing outcome scales, namely CHESS and MAPLe, allows for simple embedding as part of standard work processes. As well, the inclusion of CAPs as part of the prioritization strategy reinforces their utility for alerting the assessor to key areas of potential or actual risk. Importantly, CAPs are not intended as practice guidelines, but rather a series of empirically demonstrated strategies that lead to positive outcomes (Fries, Morris, Bernabei, Finne-Soveri, & Hirdes, 2007). At the person level, care planning with the CAP guidelines is a collaborative process with the patient (or substitute decision-maker) and family that focuses on the patient’s strengths, preferences, and needs (Gray et al., 2009). Based on this work, Central LHIN will implement a standard work process for managers and care coordinators to prioritize patients identified as high risk by focusing on the utilization of CAP guidelines for patient care planning with the aim of delaying or reducing LTC placement, hospital admissions, and preventable adverse health outcomes.

A RAI data report was developed that allows care coordinators and managers to search patient-level data for key RAI-HC outputs, including CHESS and MAPLe, by home care team and caseload. The initial benefits include the ability to quickly identify and prioritize new and existing patients who may require a more intensive care coordination approach to mitigate and address risks. For those identified at high risk for poor outcomes, the care coordinator can click a hyperlink that navigates directly to the patient’s care plan. Managers can also search a report to identify all patients at potential high risk. The report facilitates a proactive approach to complex care planning, ensuring that patients’ active care plans address their complexity and needs. This complex planning may involve the respective caregivers who may benefit from interventions (e.g., respite hours) offered by the provider. Additional benefits of the report include prioritizing patients when there is a change in caseload or care coordinator, and identifying appropriate cases for linkage to primary care programs (e.g., Health Links). At the regional and provincial level, CAP triggering rates, in addition to home care quality indicators, can be used for population need analysis to better understand local issues and performance (Morris, Fries, Frijters, Hirdes, & Steel, 2013).

### Limitations and Future Directions

It is important to acknowledge that the assumption of proportional hazards for the Cox regression was not met; however, the Akaike Information Criterion statistic (i.e., model fit) did not improve when the time by group interaction term was added. Therefore, the hazard ratio can be interpreted as the average hazard over time. A closer examination of the interaction term’s very small negative parameter estimate reveals that the hazard ratio is reliable up to about six months after the assessment at which point the hazard ratio is still at or above two. Thus, these findings are fully applicable in Ontario’s home care context in which home care patients are reassessed every six to 12 months. Additional analyses using the validation dataset also confirmed that the findings are relevant for follow-up assessments as well as intake assessments.

It is likely that this study underestimates the rates of outcomes due to the lack of information on LTC placement and death after discharge from home care – for instance, if the patient was discharged to hospital and subsequently placed into LTC. Another limitation of this study is the narrow definition of outcomes. Although LTC placement and mortality are obvious choices, some home care clients may be at heightened risk for deterioration in a broad sense (e.g., poor quality of life) but do not precipitate LTC placement or death in the short term. Other interRAI scales and algorithms may serve as good starting points, such as the Detection of Indicators and Vulnerabilities for Emergency Room Trips (DIVERT) scale for identifying patients at risk for emergency department visits (Costa et al., 2015).

## Conclusion

In summary, long-stay home care patients with high MAPLe and high CHESS are at high risk of LTC placement or death and may benefit from a comprehensive review of care plans. Since CAPs are designed to highlight areas that are amenable to clinical intervention, home care agencies are well-positioned to intervene and potentially prevent, delay, or change the course of outcomes. On a broader scale, this study affirms that factors associated with LTC placement or dying are frequently stressful and distressing, and these factors should be recognized and addressed in care planning to ensure the best quality of care for patients and their caregivers. Finally, this study is a practical example of how mobilizing the interRAI assessment system and integrating its various parts (e.g., outcome scales, algorithms, CAPs, quality indicators) can provide the critical link between assessment and action, and thus strengthen a health care organization’s commitment to provide safe, quality, and patient-focused care.
